# Vertical Transmission of H9N2 Avian Influenza Virus in Goose

**DOI:** 10.3389/fmicb.2017.01559

**Published:** 2017-08-15

**Authors:** Guanliu Yu, Aihua Wang, Yi Tang, Youxiang Diao

**Affiliations:** ^1^College of Animal Science and Technology, Shandong Agricultural University Tai'an, China; ^2^Shandong Provincial Key Laboratory of Animal Biotechnology and Disease Control and Prevention, Shandong Agricultural University Tai'an, China; ^3^Shandong Provincial Engineering Technology Research Center of Animal Disease Control and Prevention, Shandong Agricultural University Tai'an, China

**Keywords:** H9N2 AIV, LPAIV, vertical transmission, breeder goose, gosling

## Abstract

During a study on high mortality cases of goose embryo in Shandong Province, China (2014–2015), we isolated an H9N2 avian influenza virus (AIV) strain (A/goose/Shandong/DP01/2014, DP01), which was supposedly the causative agent for goose embryo death. Sequence analysis revealed that DP01 shared 99.9% homology in the HA gene with a classic immune suppression strain SD06. To study the potential vertical transmission ability of the DP01 strain in breeder goose, a total of 105 Taizhou breeder geese, which were 360 days old, were equally divided into five groups (A, B, C, D, and E) for experimental infection. H9N2 AIV (DP01) was used for inoculating through intravenous (group A), intranasal instillation (group B), and throat inoculation (group C) routes, respectively. The geese in group D were inoculated with phosphate buffer solution (PBS) and those in group E were the non-treated group. At 24 h post inoculation, H9N2 viral RNA could be detected at vitelline membrane, embryos, and allantoic fluid of goose embryos from H9N2 inoculated groups. Furthermore, the HA gene of H9N2 virus from vitelline membrane, embryo, allantoic fluid, and gosling shared almost 100% homology with an H9N2 virus isolated from the ovary of breeder goose, which laid these eggs, indicating that H9N2 AIV can be vertically transmitted in goose. The present research study provides evidence that vertical transmission of H9N2 AIV from breeding goose to goslings is possible.

## Introduction

Avian influenza virus (AIV) is a member of *Orthomyxoviridae* type A that can cause avian infectious diseases (Qiang and Diao, 2011). AIV has various subtypes, out of which H9N2 AIV was first isolated from chickens in southern China in 1994 (Guo et al., 2000). Subsequently, AIV rapidly spread in China and has become the most prevalent pathogen in poultry (Su et al., 2015). H9N2 AIV, as one of the low pathogenic avian influenza viruses (LPAIVs), causes immunosuppression in birds and is prone to induce secondary infection, decrease egg production, and cause respiratory symptoms (Nili and Asasi, 2002; Liu et al., 2003; Pantin-Jackwood et al., 2012).

According to the goose AIV epidemiological investigation in eastern China during 2014–2015, the fertility rate and hatchability of eggs from breeder birds, which were naturally infected with H9N2 AIV, significantly decreased, and H9N2 specific RNA was detected in either breeder birds or in their progenies. Previous research studies have indicated that H5N1 AIV could be vertically transmitted (Beato and Capua, 2011; Lieberman et al., 2011). As an LPAIV, H9N2 AIV may also cause H9NA AIV infection in progeny birds. However, to date no scientific study has indicated that H9N2 AIV can be vertically transmitted in goose. To clarify the route of transmission of H9N2 AIV and to effectively control the epidemic of this infectious disease, the present study was conducted to confirm H9N2 AIV as the causative agent of goose embryo death and to evaluate vertical transmission of H9N2 AIV in geese.

## Materials and methods

### Virus isolation and propagation

The DP01 isolate of H9N2 AIV (A/Goose/Shandong/DP01/2014, GenBank Accession No. MF355415) was originally isolated from dead goose embryos in Shandong Province, China. H9N2-positive dead goose embryo tissue was homogenized in PBS containing antibiotics (20% w/v) and then centrifuged at 6000 g for 30 min at 4°C. The supernatant was then filtered through a 0.22-μm syringe driven filter (Thermo Scientific, Lenexa, Kansas) and inoculated into 9-day-old SPF chicken eggs through the allantoic cavity route. Inoculated eggs were daily checked, and the allantoic fluid was harvested for virus titration at 72 h post inoculation. The median embryo lethal dose (ELD_50_) of DP01 strain was tested at 10^−7.5^/0.2 mL, which was calculated using the Reed and Muench assay (Reed and Muench, 1938).

### Experimental design

Because specific pathogen free (SPF) geese were unavailable, 360-day-old healthy Taizhou breeder geese were obtained from the Taizhou breeder goose farm (Shandong, China) for bird trail purpose. Firstly, the geese were raised for 20 days to guarantee normal egg production before inoculation. Serum samples and cloacal/tracheal swabs were then collected from breeder geese and tested by hemagglutination inhibition and RT-PCR (Cattoli et al., 2004; Xu et al., 2005) to confirm that the geese were free of H9N2 AIV infection. For experimental design, a total of 105 Taizhou breeder geese, which were 360-days-old, were randomly divided into five groups (A, B, C, D, and E), with 21 geese in each group. A 2.8-mL H9N2 AIV (DP01 strain; ELD_50_, 10^−7.5^/0.2 mL) was inoculated through intravenous (group A), intranasal instillation (group B), and throat inoculation (group C) routes. Negative control (group D) was inoculated through intravenous route with PBS. Group E was the non-treated group, which served as the blank control. All groups were separately raised in different animal facilities. The geese were reared on the floor with sterile bedding (wood shavings), where food and water were autoclaved and automatically refilled. All animal experiments were performed according to the guidelines of the Committee on the Ethics of Animals of Shandong and the appropriate biosecurity guidelines, and the protocol was approved by Shandong Agricultural University Animal Care and Use Committee. In addition, all other goose pathogens were tested negative to guarantee our experiment geese had clean background.

### Embryo sample collection

In this study, 12, 13, 10, 15, and 16 hatching eggs produced by breeder geese of groups A, B, C, D, and E, respectively, were collected. All eggs were collected for hatching, and the fertility rate and hatchability were recorded. Newly hatched goslings were raised in SPF bird isolater for further tests. The vitelline membrane, embryo, allantoic fluid, and lung (from goslings) samples from dead embryos were collected and stored at −80°C for virus detection and isolation.

### RNA extraction and RT-PCR

Viral RNA was extracted from goose embryos (i.e., vitelline membrane, embryo, allantoic fluid, and lung) using Trizol reagent (TransGen Biotech, Beijing, China) according to the manufacturer's instructions. The extracted RNA was then used for cDNA synthesizing using *TransScript*® All-in-One First-Strand cDNA Synthesis SuperMix (TransGen Biotech, Beijing, China). The primers of H9N2 AIV detection were originally designed according to conserved sequences of H9N2 strains in GenBank (Table 1). Detection gene (i.e., S4 segment, a part of NP gene) was amplified from the cDNA by PCR utilizing Cwbio EsTaq MasterMix (Cwbio, Shanghai, China), and the program was set as follows: 94°C for 5 min; 32 cycles of denaturation at 94°C for 30 s, annealing at 55°C for 35 s, and extension at 72°C for 45 s; and a final extension at 72°C for 10 min. Amplified products were separated by electrophoresis on a 0.8% agarose gel.

**Table 1:** Primers used in this study.

**Primer**	**Sequence (5′-3′)**	**Purpose**	**Products size**
S4	F: GATAGAGACTCAACCCAAAA	H9N2 AIV detection	315 bp
	R: AACATCCTTTCCCATCTTCC		
HA	F: TTCACAACCACTCAAGATGGAGACA	HA gene amplification	1671 bp
	R: CCATTGGACATGGCCCAGAA		
HA	F: GCAGTTGGTCTGAGGAATGTGCC	qRT-PCR	171 bp
	R: TTGCCTTTTGGGTTGAGTCTCTATC		

The HA gene was amplified from the cDNA by PCR using *TransScript*® DNA Polymerase High Fidelity (HiFi; TransGen Biotech, Beijing, China) with amplification primers (Table 1), which were also designed following conserved sequences of H9N2 strains in GenBank. The PCR program was set as follows: 94°C for 5 min; 32 cycles of denaturation at 94°C for 40 s, annealing at 55°C for 50 s, and extension at 72°C for 2 min; and a final extension at 72°C for 10 min. PCR products were then purified, cloned into pMD18-T vector (TaKaRa, Dalian, China), and sequenced using the Sanger sequencing assay (TsingKe Biotech, Beijing, China).

### Quantitative analysis of virus RNA concentration

H9N2 viral RNA concentration was measured from fallopian tube and ovarian tissues in breeder goose infected with H9N2 AIV at 1, 3, 5, and 7 dpi using quantitative SYBR Green I real-time PCR (qRT-PCR) with the 7500 Fast Real-Time PCR System (Applied Bio systems, CA, USA). The primers were designed following conserved sequences of H9N2 strains in GenBank (Table 1). For confirming H9N2 AIV copy numbers in the above tissues, viral RNA concentration (log_10_) were normalized per 1 μg of total RNA. Furthermore, qRT-PCR was performed using the SYBR Green PCR kit (TransGen, Beijing, China) in a total volume of 25 μl following the product instructions. The amplification program was set as follows: 95°C for 5 min; 40 cycles of denaturation at 94°C for 15 s and 60°C for 34 s; and a final melting curve analysis step. Each sample was analyzed in triplicates.

### Histopathology observation

The dead breeder geese infected by H9N2 AIV were dissected, and fallopian tube and ovaries were collected in 10% neural buffered formalin. Tissues were sectioned in paraffin with 4-mm thickness and stained with hematoxylin and eosin using standard methods. Finally, histopathological changes were observed under a microscope.

### Phylogenetic analysis

Nucleotide sequence analysis was performed based on the complete HA gene of H9N2 AIV. Sequences from the newly isolated strains (from vitelline membrane, embryo, allantoic fluid, and goslings) were compared to the original DP01 isolate. The alignment was constructed using the ClustalW method of MegAlign program of DNA Star software (Doan et al., 2004). Phylogenetic comparisons were conducted using neighbor-joining analysis by MEGA 5.05 software (Tamura et al., 2011), where bootstrap confidence values were determined using 1,000 replicates.

### Statistical analysis

SAS 9.0 software (SAS Institute, Inc., Cary, NC, USA) was used for statistical analysis, and data are expressed as mean ± *SD*; one-way ANOVA with multiple range test was used to compare the difference of viral titers among groups, where *P* < 0.05 or *P* < 0.01 represented statistical significance.

## Results

### Epidemiological investigation

**Figure 1:**
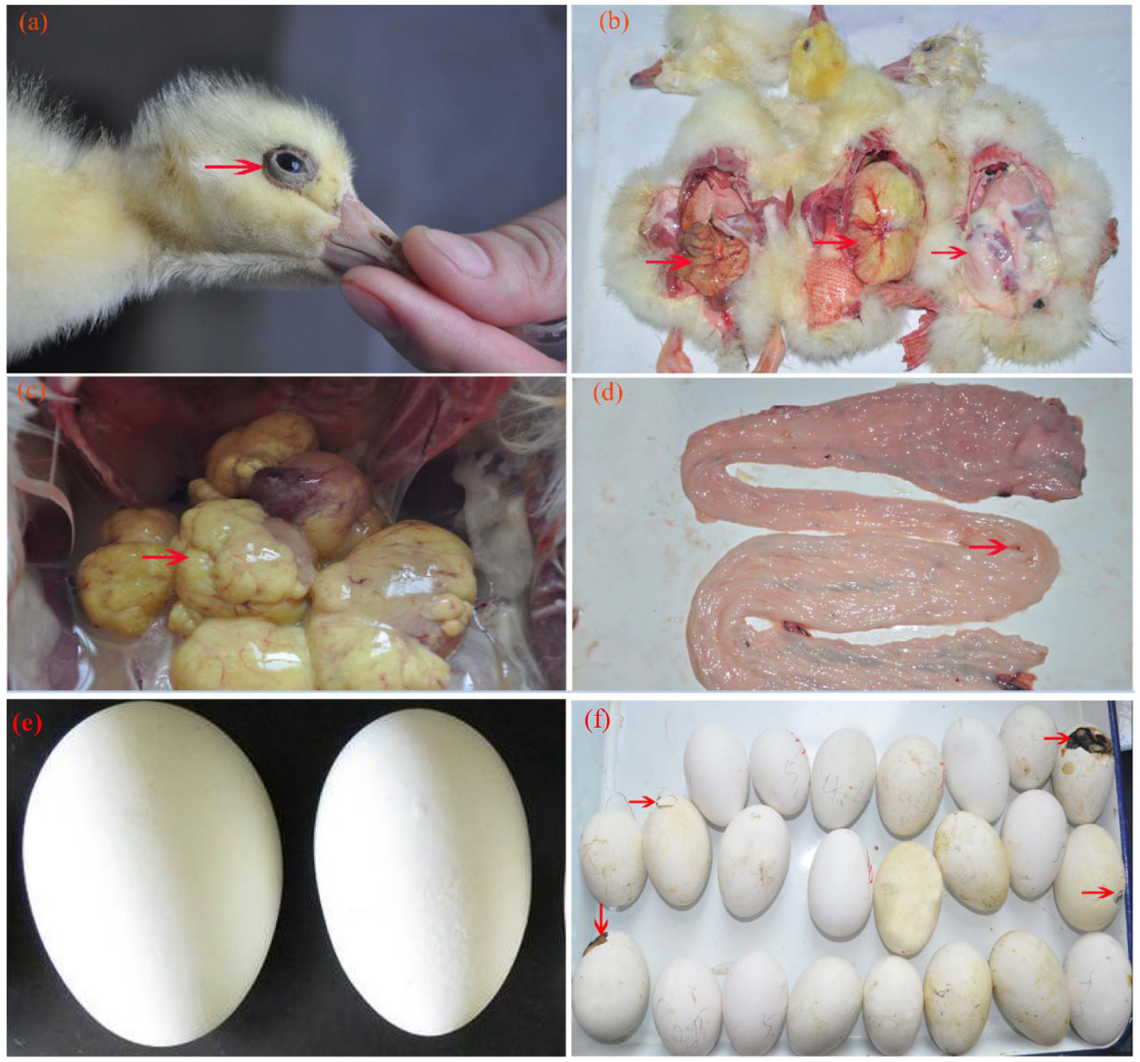
Clinical symptoms and pathological changes of breeder goose infected with H9N2 AIV. **(a)** Swollen eyes with tears; **(b)** yolk was not absorbed completely; **(c)** deforming of ovarian follicles, liquidation and hemorrhage (group A); **(d)** fallopian tube edema, rarely seen hemorrhagic spots (group C); **(e)** various sizes of breeder eggs [light: normal egg; right: infected eggs, sand shell egg (group A)]; **(f)** dead gosling failed to break the shells out.

In 2014–2015, we conducted an AIV epidemiological investigation on goose in eastern China. Egg production drop, sand shell egg, failed capability of embryos to break the shells out (Figure 1f), and decreased fertility rate and hatchability were observed in affected geese. Positive rates of H9N2 AIV in naturally infected samples are shown in Table 2 (as measured by RT-PCR).

**Table 2:** Positive rate of H9N2 AIV in epidemiological investigation (as estimated by RT-PCR).

**Positive rate (No. positive/no. tested)**				
**Breeder goose**	**Breeder eggs**	**Goose embryos**	**Goslings**	**Total**
21.9% (16/73)	33.3% (22/66)	18.2% (4/22)	12.1% (7/58)	22.4% (49/219)

### Clinical signs and gross lesions following inoculation of H9N2 AIV in geese

**Figure 2:**
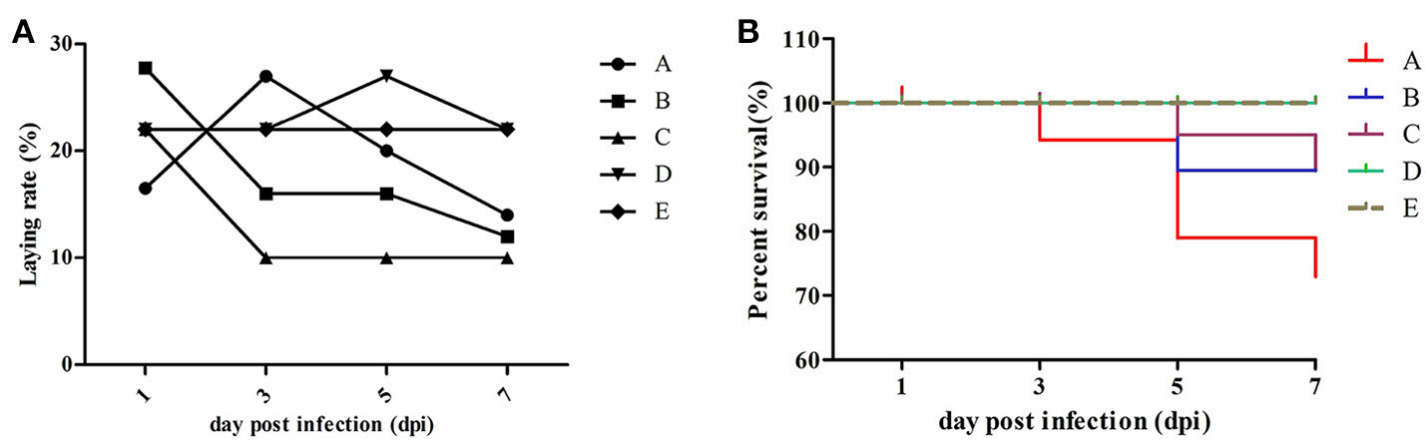
Laying rate **(A)** and survival curve **(B)** of different groups. Data were processed by GraphPad Prism 5.0 (GraphPad Software Inc., SanDiego, CA, USA).

Approximately half of the geese in group A showed diarrhea and reduced appetite at 1 dpi, while three dead geese were found at 3 dpi first. In group B, four dead geese were found at 5 dpi, and the most remarkable characteristic was watery eyes and edema that appeared in groups A and B at 9 dpi. In group C, two dead geese were found at 5 dpi, and some geese exhibited slight respiratory distress at 7 dpi; however, approximately half of them showed reduced respiratory distress signs at 9 dpi. No dead geese were found in groups D and E. Importantly, newly hatched goslings exhibited swollen eyelids and watery eyes (Figure 1a); the yolk of dead goslings were not completely absorbed and were hemorrhaged (Figure 1b). Ovarian tissues of dead breeder goose in group A exhibited liquidation, deformation, and hemorrhage (Figure 1c), whereas fallopian tube edema was found in group C (Figure 1d). In addition, no clinical signs or gross lesions were found in control groups. Survival curves of above groups are shown in Figure 2B.

### Reduced Egg production and viability in H9N2 AIV infected geese

As shown in Figure 2 and Table 3, laying rates were generally higher in control groups (i.e., D and E) than in infected groups (i.e., A, B, and C). A total of 35 hatching eggs produced by H9N2 AIV infected goose from infected groups and 31 produced by goose from control groups were collected and incubated. Importantly, some sand eggs were found in group A whose size was significantly smaller in this group than in control groups (Figure 1e). Statistical data showed that hatchability was higher in control groups than in infected groups (Table 3).

**Table 3:** Fertilization rate and hatchability of hatching eggs samples.

**Groups**	**Fertility rate (no. fertilized/total)**	**Hatchability (no. hatched/fertilized)**
A	50.0%(6/12)	0.0%(0/6)
B	69.2%(9/13)	11.1%(1/9)
C	70.0%(7/10)	14.3%(1/7)
D	86.7%(13/15)	76.9%(10/13)
E	81.3%(13/16)	84.6%(11/13)

### Detection of viral RNA in infected geese and embryos Following inoculation of H9N2 AIV

RT-PCR amplified products were detected by electrophoresis on a 0.8% agarose gel, and electrophoresis results corresponded with the amplified length of target gene. As shown in Table 4, six (50.0%) of 12 vitelline membrane samples, 10 (43.5%) of 23 embryo samples, eight (72.7%) of 11 allantoic fluid samples, and two (100.0%) of two lung samples were found positive for H9N2 AIV, and the nature of the PCR product was confirmed by sequencing (see below). In contrast, RT-PCR results were negative in control groups.

**Table 4:** Positive rate of H9N2 AIV in samples as tested by RT-PCR.

**Groups**	**Positive rate (no. positive/ no. tested)**			
	**Vitelline membrane**	**Embryo**	**Allantoic fluid**	**Lung**
A	80.0% (4/5)	42.9% (3/7)	75.0% (3/4)	–
B	25.0% (1/4)	44.4% (4/9)	75.0% (3/4)	100.0% (1/1)
C	33.0% (1/3)	42.9% (3/7)	66.7% (2/3)	100.0% (1/1)
D	0.0% (0/5)	0.0% (0/8)	0.0% (0/4)	–
E	0.0% (0/7)	0.0% (0/5)	0.0% (0/9)	–

**Figure 3:**
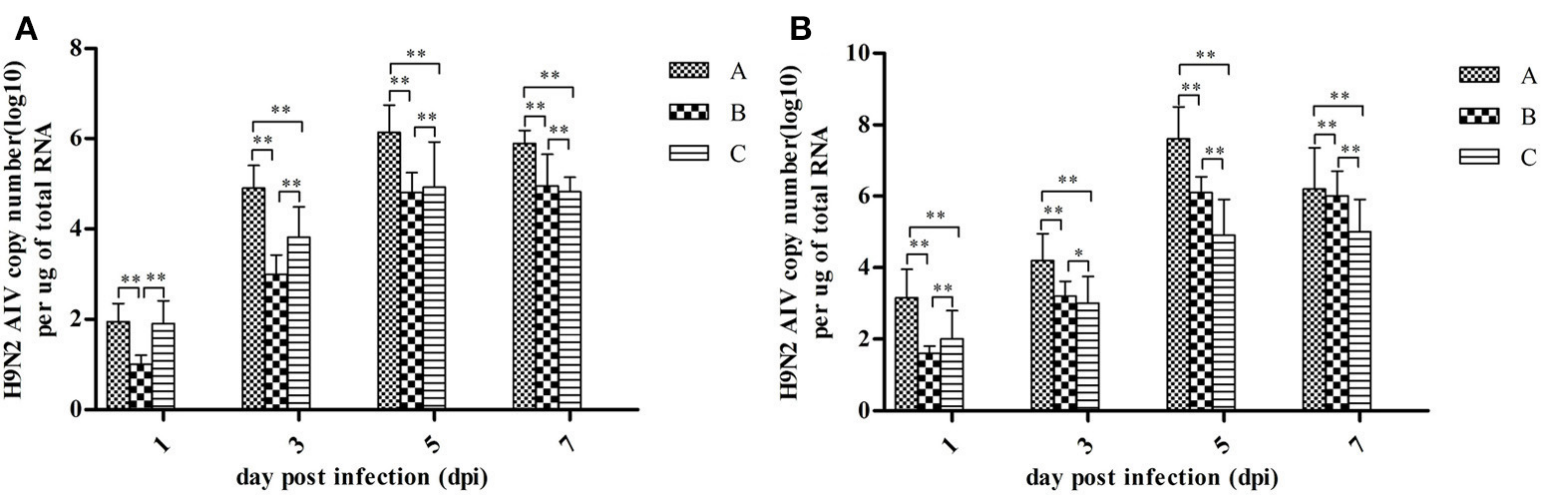
H9N2 AIV viral RNA concentration in fallopian tubes **(A)** and ovaries **(B)** at different dpi. Data are expressed as mean ± *SD* (*n* = 3) and processed by GraphPad Prism 5.0 (GraphPad Software Inc., SanDiego, CA, USA). ^**^P < 0.01, ^*^P < 0.05. Significant differences were calculated by One-way ANOVA with Duncan's multiple range test (SAS Institute, Inc., Cary, NC, USA).

Quantitative measurements of viral RNA concentration revealed highest levels at 5 dpi, with a slight decrease at 7 dpi (see Figure 3). Notably, the concentration of viral RNA was higher in group A than in Groups B and C (*P* < 0.01). Viral RNA was not detected in control groups (data not shown).

### Histopathology observation

**Figure 4:**
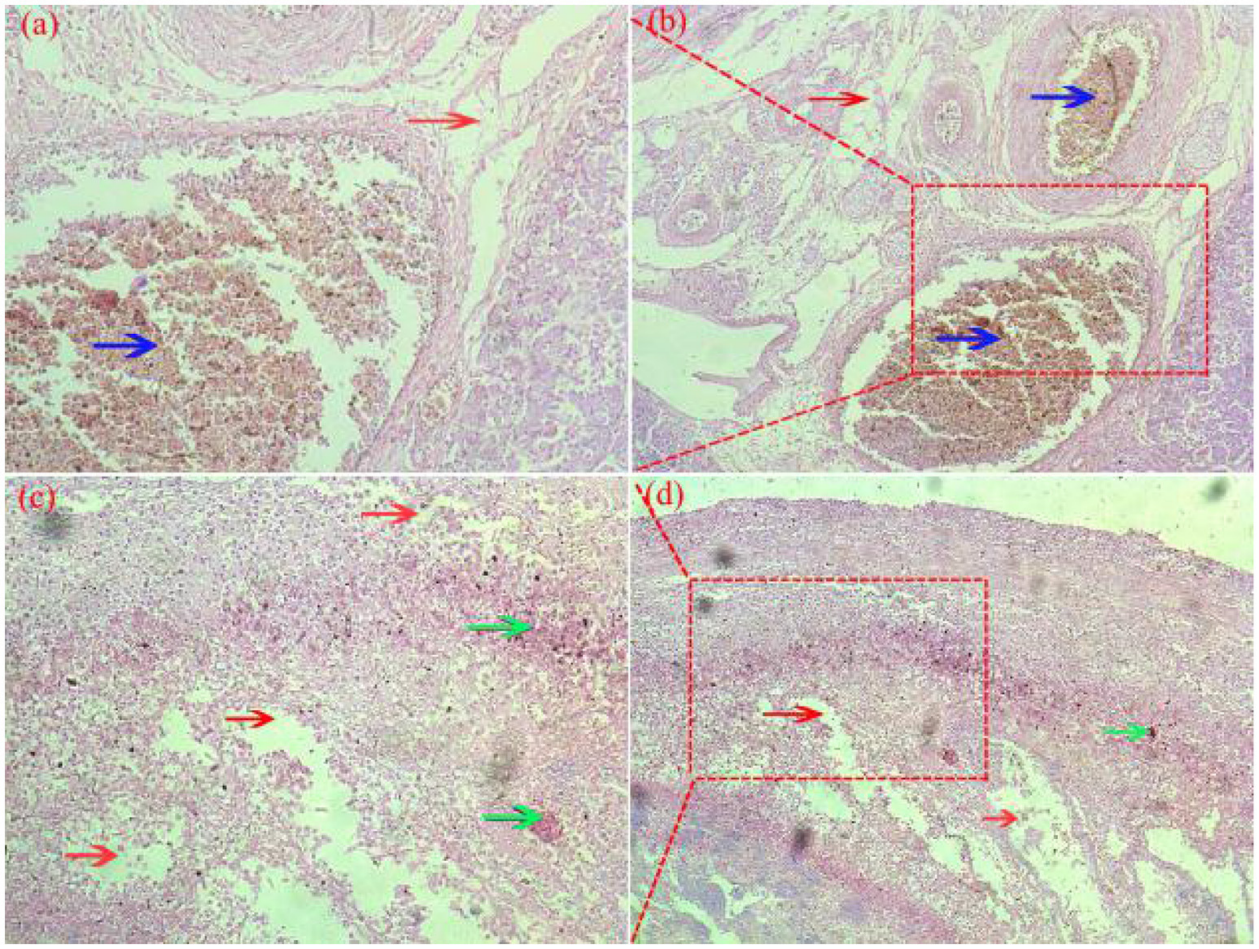
Pathological changes of ovaries **(a,b)** and fallopian tubes **(c,d)** of H9N2-infected breeder goose (7dpi, Group A). Theca follicle edema (red arrow on **a,b**) with massive hemorrhage (blue arrow on **a,b**), fallopian tubes mucous layer cell falls off, hemorrhage (blue arrow on c and d), lymphocytes infiltration (green arrow on **c,d**) and edema (red arrow on **c,d**). Magnification, **(a,c)** shown at 100x, **(b,d)** shown at 200x.

Pathological changes were detected in ovarian and fallopian tubes of breeder goose infected with H9N2 AIV: theca follicle edema with massive hemorrhage (Figures 4a,b), fallopian tubes mucous layer cell falling off, along with hemorrhage and edema (Figures 4c,d). In contrast, no pathological changes were found in control groups.

### Sequence comparison of HA gene

Sequence analysis of the HA gene revealed 99.9% sequence identity (at the nucleotide level) between the DP01 isolate and the SD06 strain (Qiang and Diao, 2011).

**Figure 5:**
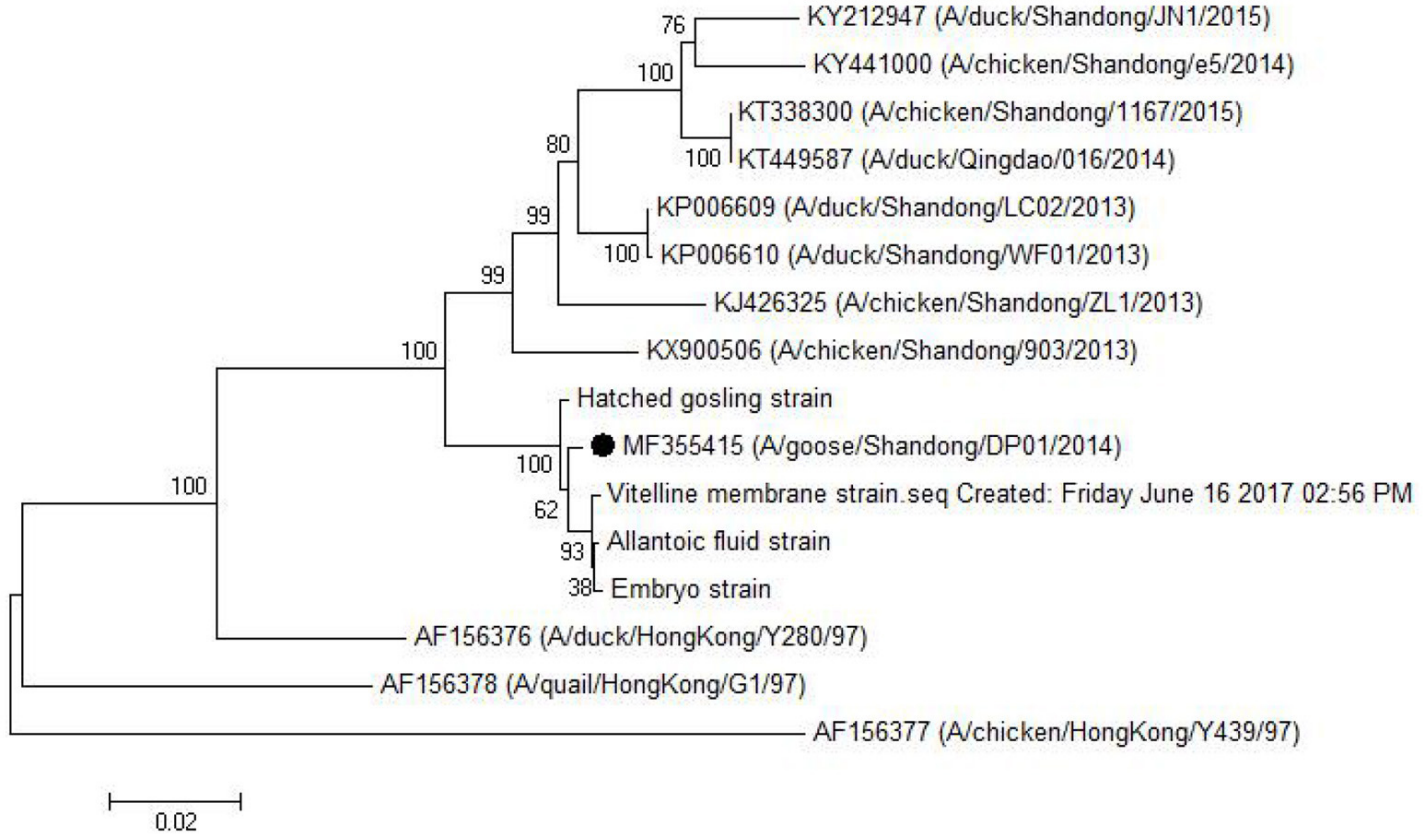
Phylogenetic tree comparing the HA gene nucleotide sequence of the DP01 isolate with that of other strains and isolates. The sequence corresponding to the virus isolated at DP01 is marked by a black dot. The phylogenetic tree was built using Neighbor-Joining analysis by MEGA 5.05 software, and the bootstrap confidence values were determined using 1,000 replicates.

As shown in Table 5, the HA gene amplified from viral RNA isolated from vitelline membrane, embryo, allantoic fluid, and goslings, shared 99.3–99.9% nucleotide and 99.3–99.7% amino acid sequence identity to that of the DP01 isolate, implying that it was the same strain that was transmitted vertically. Compared with other AIV strains in Shandong Province, the DP01 isolate shared 93.0–95.2% nucleotide and 93.8–96.8% amino acid sequence identity with chicken-isolated strains and 93.1–95.4% nucleotide and 94.1–96.7% amino acid sequence identity with duck-isolated strains. Notably, all sequences retrieved from this study grouped with Y280-like sequences (Figure 5).

**Table 5:** Sequence identity amongst viruses isolated in this study and with previously identified virus isolates.

		**% Nucleotide identity**															
**% Amino acid identity**		**1**	**2**	**3**	**4**	5	6	7	8	9	10	11	12	13	14	15	16
**1**	**DP01 isolate**	**100**	**99.9**	**99.8**	**99.8**	**99.3**	94.4	95.2	93.0	94.5	95.4	95.3	94.1	93.1	92.1	87.8	82.3
**2**	**Virus isolated from allantoic fluid**	**99.7**	**100**	**99.8**	**99.8**	**99.3**	94.4	95.2	93.0	94.5	95.4	95.3	94.1	93.1	92.1	87.8	82.3
**3**	**Virus isolated from embryos**	**99.5**	**99.5**	**100**	**98.7**	**99.3**	94.6	95.2	93.2	94.7	95.5	95.5	94.3	93.2	92.2	87.8	82.3
**4**	**Virus isolated from vitelline membrane**	**99.5**	**99.5**	**99.3**	**100**	**99.3**	94.4	95.2	92.9	94.5	95.4	95.3	94.1	93.1	92.1	87.7	82.3
**5**	**Virus isolated from gosling**	**99.5**	**99.5**	**99.3**	**99.3**	**100**	94.9	95.7	93.2	94.9	95.7	95.6	94.5	93.7	92.5	88.2	82.7
6	KJ426325 (CK/SD/ZL1)	95.0	95.0	95.5	94.8	95.5	100	95.7	94.4	96.3	96.9	96.8	95.7	94.6	90.9	86.7	81.0
7	KX900506 (CK/SD/903)	96.8	96.8	97.0	96.6	97.3	95.5	100	93.9	95.6	96.1	96.0	95.2	94.4	91.6	87.2	81.4
8	KY441000 (CK/SD/e5)	93.8	93.8	94.3	93.6	94.3	93.6	94.8	100	97.5	95.8	95.8	97.6	96.7	89.7	86.8	81.2
9	KT338300 (CK/SD/1167)	95.0	95.0	95.5	94.8	95.5	94.8	96.1	97.7	100	97.5	97.4	98.8	97.6	91.1	87.5	81.3
10	KP006609 (DK/SD/LC02)	96.7	96.7	97.2	96.6	97.2	96.6	97.3	95.9	97.1	100	99.9	97.0	95.9	91.3	87.3	81.8
11	KP006610 (DK/SD/WF01)	96.6	96.6	97.1	96.4	97.1	96.4	97.1	95.7	97.0	99.8	100	96.9	95.8	91.3	87.2	81.7
12	KT449587 (DK/QD/016)	94.1	94.1	94.7	93.9	94.7	93.9	95.2	97.7	98.2	96.3	96.1	100	97.4	90.7	87.2	81.2
13	KY212947 (DK/SD/JN1)	94.3	94.3	94.5	93.9	94.8	93.9	95.4	97.5	97.7	96.1	95.9	97.7	100	90.0	86.6	81.0
14	AF156376(DK/HK/Y280/97)	93.9	93.9	94.1	92.5	94.4	92.5	93.7	91.4	92.3	93.5	93.3	91.6	91.4	100	90.6	83.8
15	AF156378 (Quail/HK/G1/97)	89.7	89.7	89.9	87.5	90.2	87.5	89.0	88.0	88.8	88.6	88.4	88.2	88.0	90.6	100	84.7
16	AF156377 (DK/HK/Y439)	87.3	87.3	87.5	85.6	87.9	85.6	96.2	85.0	85.4	86.2	86.0	85.2	84.8	89.3	88.5	100

*The bold values was to highlight the high homology of the original strain (DP01) and the newly isolated strains*.

## Discussion

H9N2 AIV was initially isolated in turkey flocks in North America (Homme and Easterday, 1970). It has been an endemic and established stable lineage in several countries and worldwide since 1990s (Ge et al., 2009). At present, H9N2 is one of the major subtypes of AIV circulating in several parts of the world, which poses a great threat to poultry industry and public health (Pantin-Jackwood et al., 2012; Gu et al., 2015). To effectively control the spread and epidemic of this infectious disease, it is necessary to understand the route of H9N2 AIV transmission. In 2014–2015, we conducted an AIV epidemiological investigation on goose in eastern China, and obtained evidence for decreased fertility rate and hatchability in affected geese. We also observed major pathological changes of oviduct caused by H9N2 AIV infection in birds: falling epithelial villus or epithelial cell necrosis in magnum and broad hyperemia and hemorrhage in tissue of shell gland (Wang et al., 2013). Taken together, these findings suggest that H9N2 AIV might be vertically transmitted in birds.

To the best of our knowledge, this is the first study that indicates that H9N2 AIV can be vertically transmitted in breeder goose. In this study, we infected experimental geese with H9N2 AIV through different inoculation routes: intravenous (group A), intranasal instillation (group B), and throat inoculation (group C). As shown in Table 4, the total H9N2 AIV positive rate in goose embryos was highest in group A (62.5%, 10/16) compared with groups B (41.7%, 8/17) and C (46.1%, 6/13). This may be caused by a more effective viral dose for cell infection, which depends on various inoculating routes. For group A, virus particles were directly transferred to different tissues or organs (i.e., ovary or fallopian tube) through blood circulation as viremia and caused the systemic infection. For groups B and C, because of the protection of respiratory mucosa and cilia in respiratory tract, the virus infection was like delayed, reducing the rates of systemic infection.

In the present study, viral RNAs were detected from fallopian tube and ovarian tissues in breeder goose infected with H9N2 AIV at 1, 3, 5, and 7 dpi. Notably, the concentration of viral RNA reached a peak at 5 dpi (Figure 3), which resulted in the decline of viral RNA concentration at 7 dpi. The main pathological changes were found in reproductive organs of breeder geese and included exudative liquefied and hemorrhaged ovaries (Figures 1c, 4a,b) and edema and hemorrhage in follicles tube (Figures 1d, 4c,d). This might be due to the presence of high concentration of SAα-2 and 3GAL receptors in the genital tracts of geese (Wang et al., 2013), which can bind HA protein of AIV and induce more severe infection (Connor et al., 1994; Zhang et al., 2010).

H9N2 AIV can be divided into Northern America and Eurasian strains. The Eurasian strains were also divided into three groups as follows: the I lineage represented by A/Duck/Hong Kong/Y280/97 (Y280-like), the II lineage represented by A/Duck/Hong Kong/Y439 (Y439-like), and the III lineage represented by A/Quail/Hong Kong/G1/97 (G1-like; Lin et al., 2000). As previously reported with regard to the pathogenesis of Y280-like lineage, infected chickens or ducks usually exhibit respiratory symptom, eggs drop, anorexia, etc. (Zhang et al., 2008; Bi et al., 2010; Qiang and Diao, 2011; Huang et al., 2013). However, in this study, infected geese showed only slight respiratory distress, although the damage of genital system was relatively serious. Hence, to explore the difference in the pathogenesis of different hosts, we compared the HA gene from the newly isolated viruses from vitelline membrane strain and embryos to that of the Shandong (four chicken-isolated strains and four duck-isolated strains in Shandong Province), and Eurasian (Y280-like, G1-like, and Y439-like) representative strains. As shown in Table 5 and Figure 5, the isolates examined in this study had a very close relationship to the Shandong-isolated and Y280 strains. Identifying the determinants for the different pathogenesis will be the subject of further study.

To summarize, our findings provided adequate evidence that H9N2 AIV is vertically transmitted from breeding goose to goslings.

## Author contributions

GY and AW completed most of the experiments. YT and YD designed experiments and reviewed the manuscript.

### Conflict of interest statement

The authors declare that the research was conducted in the absence of any commercial or financial relationships that could be construed as a potential conflict of interest.
